# Knowledge of obstetrical fistula and its associated factors among reproductive-age women in Ejere Woreda, West Shewa Zone, Oromia Region, Ethiopia: a community-based study

**DOI:** 10.3389/fgwh.2025.1547599

**Published:** 2025-08-29

**Authors:** Getahun Tiruye, Daba Kejela, Anteneh Dirar, Abel Tibebu Goshu, Teklemariam Gultie

**Affiliations:** ^1^ Hamlin College of Midwives, Addis Ababa, Ethiopia; ^2^ College of Health and Medical Science, Haramaya University, Harar, Ethiopia; ^3^ Institute of Health, Jimma University, Jimma, Ethiopia; ^4^ Menelik II Medical and Health Science College, Addis Ababa, Ethiopia; ^5^ College of Health Science, Arba Minch University, Arba Minch, Ethiopia

**Keywords:** knowledge, reproductive-age women, obstetric fistula, Ejere, Ethiopia

## Abstract

**Introduction:**

Obstetric fistula is a serious and tragic childbirth injury that mainly affects women in resource-limited areas, often leading to social isolation and stigma. While optimal knowledge among women about the prevention, contributing factors, and available treatments for obstetric fistula is crucial for reducing its overall burden, many women in Ethiopia continue to be affected by it and endure its consequences because of a persistent knowledge gap. Therefore, this study aimed to assess the level of knowledge about obstetric fistula and its associated factors among women of reproductive age in Ejere Woreda, West Shewa Zone, Oromia Region, Ethiopia.

**Methods:**

A community-based cross-sectional study was conducted among reproductive-age women in Ejere Woreda, West Shewa Zone, Ethiopia, from 1 to 30 June 2024. A systematic random sampling technique was employed to recruit 770 participants. Data were collected using a pretested structured questionnaire, which was then coded and analyzed using SPSS version 27. Variables with a *p*-value <0.25 in bivariate logistic regression were included in a multivariable logistic regression to identify significant predictors of women's knowledge about obstetric fistula. A *p*-value <0.05 at a 95% confidence interval (CI) was used to determine statistically significant associations.

**Results:**

The study found that 46.6% (95% CI: 43.1–50.3) of women had good knowledge about obstetric fistula. Factors significantly associated with the knowledge level of obstetric fistula included being an urban resident [adjusted odds ratio (AOR) = 4.12, 95% CI: 2.36–7.19], access to a TV/radio at home (AOR = 2.51, 95% CI: 1.19–5.25), proximity to health facilities (AOR = 4.88, 95% CI: 2.37–10.04), giving birth at health institutions (AOR = 4.62, 95% CI: 2.56–8.33), attending pregnant women's conferences (AOR = 3.42, 95% CI: 1.88–6.22), and having a history of modern contraceptive use (AOR = 4.82, 95% CI: 2.77–8.37).

**Conclusion and recommendations:**

Nearly one in two women of childbearing age are knowledgeable about obstetric fistula. The study underscores the need to address the urban–rural disparity in healthcare access and information, enhancing media access, and promoting women's participation in pregnancy conferences to enhance knowledge of obstetric fistula.

## Introduction

Obstetric fistula (OF) is one of the most serious and tragic childbirth injuries and a significant cause of maternal morbidity (1). Globally, an estimated 50,000–100,000 women are affected by OF each year (2). Although advances in medical infrastructure and access to timely obstetric care have led to the near eradication of obstetric fistula in high-income countries, it continues to pose a serious public health challenge in many low-income regions, particularly in sub-Saharan Africa (SSA) and Southeast Asia (3). In these areas, access to quality maternal healthcare is often limited or delayed, leading to preventable complications during childbirth. Conservative estimates suggest that over 2 million young women are currently living with untreated OF, many of whom suffer in silence due to stigma and lack of awareness. In SSA alone, an estimated 30,000–130,000 new cases of obstetric fistula occur each year, accounting for more than 60% of the global disease burden (1, 2).

In response to this ongoing crisis, several global organizations, including the United Nations Population Fund (UNFPA), the Campaign to End Fistula, and the annual observance of the International Day to End Obstetric Fistula, have launched extensive initiatives aimed at prevention, treatment, and public education (4, 5). These efforts also align with the broader global agenda of achieving the third Sustainable Development Goal (SDG 3), which focuses on ensuring healthy lives and promoting wellbeing for all at all ages, with a particular focus on improving maternal and reproductive health (6).

In Ethiopia, where the fertility rate remains high (7), teenage pregnancy is prevalent (8), cultural malpractice exists (9), and only half (49.8%) of the births are attended by skilled birth attendants, an estimated 31,000 women are still living with untreated fistula injuries in 2022 (10).

Obstetric fistula is a severe maternal health condition predominantly affecting women in low-resource settings, including Ethiopia. It leaves women with few options for earning a living, which exacerbates their poverty (3). Women suffering from OF are abandoned by their spouses and face social stigma, which could lead to low self-esteem, despair, and long-term emotional trauma (11, 12). Women who are victims often experience severe mental health issues, including depression and anxiety, resulting from stigma and social isolation (13). Furthermore, obstetric fistula can cause long-term health problems such as kidney disease, nerve damage, leg ulcers, and, in extreme situations, paralysis (3).

Available evidence indicates that major risk factors for OF include early marriage, rural residence, and poverty. Furthermore, a large proportion of OFs is attributed to a lack of knowledge about its prevention and management (14).

The Ethiopian government has implemented several strategies to address OF, including reducing adolescent pregnancies, improving access to obstetric care, raising awareness about its complications, and introducing appropriate treatment methods (15). The previous national strategic plan aimed to reduce the number of women affected by OF to fewer than 1,600 by 2020; however, this goal was not achieved. The current national strategy (2021–2025) aims to reduce the incidence from 953 (0.03%) to 520 cases (0.016%). However, despite ongoing interventions, approximately 1,000 new cases are reported each year, keeping the overall burden relatively unchanged (10).

In addition to improving the availability of comprehensive emergency obstetric and neonatal care and ensuring the quality of maternity services, increasing public awareness through community engagement is crucial to significantly reduce both the occurrence and long-term impact of OF. Nationally, only 39% of the population, including women, has an adequate understanding of obstetric fistula. In the Oromia Region, where our study area, Ejere Woreda, is located, awareness drops further to just 28.6%, which is considerably lower than in Tigray (65.8%) and Amhara (45%) (16, 17). Ejere Woreda, in the West Shewa Zone of Oromia, faces challenges common to many Ethiopian rural districts, such as limited access to maternal health services, high rates of home births, early marriage, adolescent pregnancy, and weak referral systems that further delay access to emergency obstetric care. These factors collectively increase the risk of fistula and hinder timely medical intervention.

Studying obstetric fistula and its associated factors among women of reproductive age is vital from a medical perspective, as this group is most likely to experience pregnancy and, consequently, faces a higher risk of developing obstetric fistula due to childbirth complications, especially in regions with limited access to emergency obstetric care. Understanding the prevalence and risk factors of OF in this population is crucial for preventing unnecessary suffering and enabling more effective targeting of interventions. Such knowledge empowers the most at-risk group to prevent the condition, seek care early, reduce stigma, and participate in informed decision-making. It also informs public health strategies and supports global efforts to improve maternal health and promote gender equity.

Having adequate knowledge about the causes, risk factors, and treatment options for OF can help women protect themselves from the condition. However, there is a notable gap in research on women's knowledge of OF, particularly in Ethiopia and in the specific study area, despite the significant burden. Therefore, this study aims to assess the level of awareness and identify factors associated with knowledge of obstetric fistula among reproductive-age women in Ejere Woreda, West Shewa Zone, Oromia Region, Ethiopia.

## Materials and methods

### Study area and period

The study was conducted in Ejere Woreda from 1 to 30 July 2024. It is one of the woredas in the West Shewa Zone of Ethiopia's Oromia Region, located 45 km from the national capital, Addis Ababa. In Ethiopia, woreda is an Amharic term commonly translated as district, representing the third tier in the country's administrative hierarchy, after regions and zones. Each woreda is further divided into smaller units called kebeles, which function as the most basic level of local government in Ethiopia. The administrative center of the woreda is Addisalem Ketema. It is divided into 26 rural and 5 urban kebeles. According to the 2023 Ejere Woreda Health Office report, the overall population is estimated at 140,161, with 84,121 males and 56,040 females, including 28,739 reproductive-age women.

### Study design

A community-based cross-sectional study design was employed.

### Source population

All reproductive-age women (15–49 years) living in Ejere Woreda were considered the source population, while all reproductive-age women living in randomly selected kebeles of Ejere Woreda during the study period comprised the study population.

### Inclusion and exclusion criteria

Women of reproductive age who had been living in Ejere Woreda for at least 6 months and were registered in the health extension program folder were included in the study, while those who were severely ill and unable to respond were excluded.

### Sample size determination and sampling procedure

The sample size was determined using a single population proportion formula, considering the knowledge level of OF among reproductive-age women in Ethiopia, which was 36.4% (18), a 95% confidence level, a 5% marginal error, a design effect of two, and a 10% non-response rate.

Using the formula, the sample size is calculated as follows:

$$\;N=(Z\alpha /2{)}^{2}\times \;P\;(1-P)/d^{2}$$

$$\begin{array}{rl} & \;\mathrm{where}\;N=\mathrm{Desired}\;\mathrm{sample}\;\mathrm{size}\\ & \;Z=1.96\;\mathrm{for}\;95\text{\%}\mathrm{confidence}\;\mathrm{level}\\ & \;P=\mathrm{Knowledge}\;\mathrm{level}\;\mathrm{of}\;\mathrm{OF}\\ & \;d=\mathrm{Degree}\;\mathrm{of}\;\mathrm{precision}=0.05 \end{array}$$

$$N=\frac{\left(1.96)(1.96)(0.364)(0.636\right)}{\left(0.05)(0.05\right)}=356\times 2\;(\mathrm{design}\;\mathrm{effect})=712$$

After adding a 10% non-response rate, the final sample size was 784.

A multistage sampling technique was employed to select study participants. From the total 31 kebeles in Ejere Woreda (26 rural and 5 urban), 7 kebeles (1 urban and 6 rural) were selected using a simple random sampling method. Then, from all these kebeles, a list of households with women of reproductive age was obtained from the health extension workers’ registration book (family folders), which included their respective addresses and townhouse numbers. The family folders are continuously updated through collaboration among kebele administrative authorities and health extension workers.

**Figure 1:**
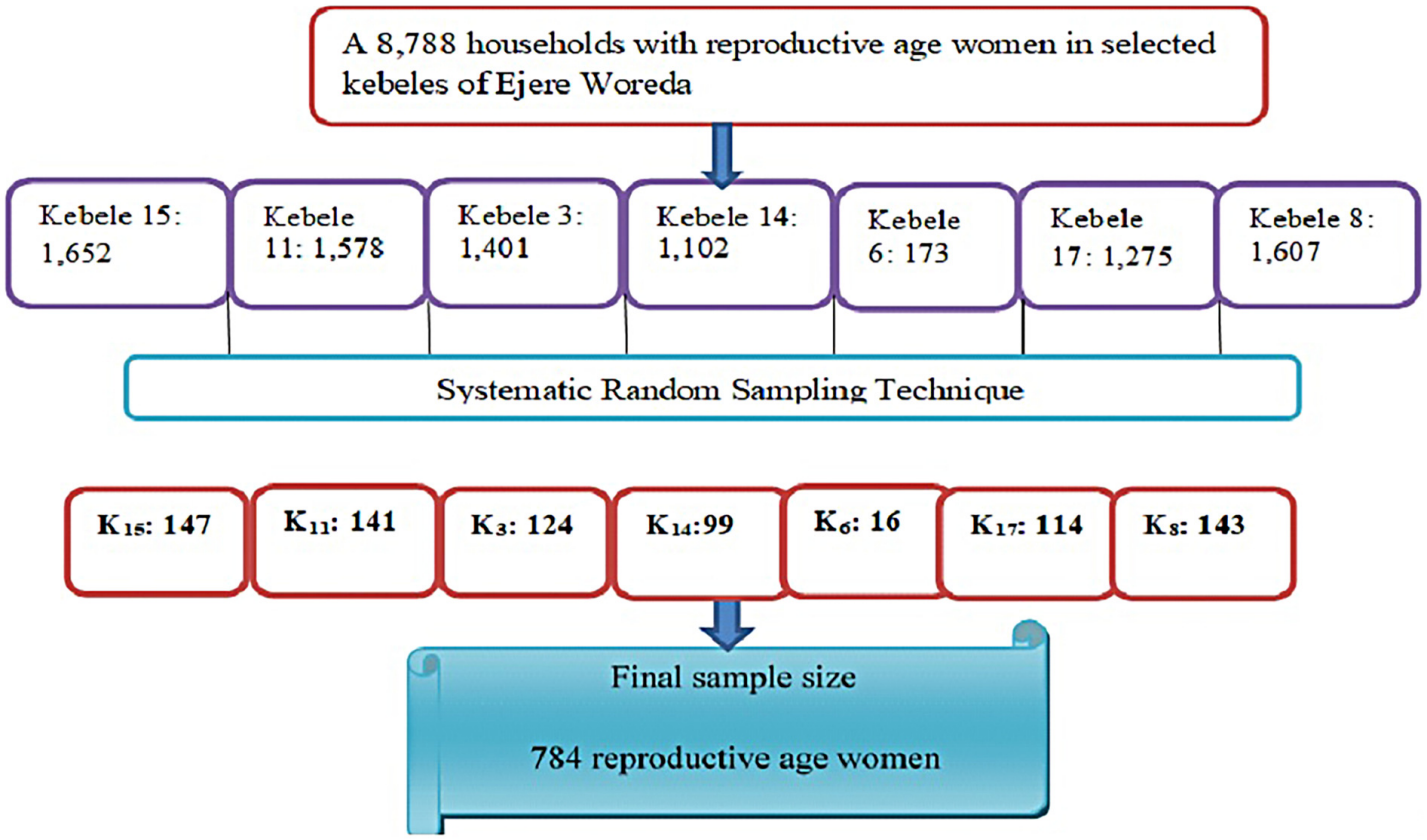
Schematic presentation of sampling procedure on seven kebeles, among reproductive-age women in Ejere Woreda, West Shewa Zone, Oromia Region, Ethiopia, 2024 (*n* = 784).

There were 8,788 reproductive-age women in the selected kebeles. Following proportional allocation based on the kebele-specific counts of eligible women, a systematic sampling technique with a *k*th interval (8,788/784) was employed to select study participants within each kebele. The first household was selected by a lottery method, and subsequent reproductive-age women were recruited at regular intervals (every “*k*th” woman). In households with more than one eligible woman, the study employed a random selection process using the lottery method to ensure that only one participant per household was included (Figure 1).

### Operational definition

#### Knowledge of OF

In this study, “knowledge of OF” refers to mothers’ understanding of the risk factors, signs and symptoms, availability of treatment, and preventive methods for OF. Comprehensive knowledge of OF was measured using 10 questions containing 29 items, which mainly comprised whether participants had ever heard of OF, knew the types of OF, its causes/risk factors, signs and symptoms, available treatments, prevention methods, and obstetric complications. Responses for each item were scored as “1” for a correct answer and “0” for a wrong answer. The scores were then summed, and the mean score was computed to be 9.27. Finally, women who scored above the mean were categorized as having “good knowledge of OF,” while those who scored below the mean were categorized as having “poor knowledge of OF” (18).

### Data collection tools and procedures

A pretested, interviewer-administered questionnaire was used to collect data from the study participants. The questionnaire was designed in English, translated into Afan Oromo (the locally spoken language), and then back-translated into English to ensure consistency. It was adapted and contextualized into the local setting from the reviewed literature (19–23). The questionnaire contains three parts. The first and second parts assessed the socio-demographic characteristics of the study participants and obstetric-related characteristics, respectively. The third part evaluated women’s knowledge of OF. Four trained health extension workers and one BSc nurse collected data and supervised the data collection process, respectively.

### Data quality assurance

The data collection process was closely monitored, with the supervisor and then the principal investigator checking the collected data daily for any incomplete content. A pretest of the questionnaire was conducted on 5% of the sample (40 reproductive-age women) outside the study area, in Ammaro kebeles, 2 weeks before the data collection period, to check the response, language clarity, and the appropriateness of the questionnaire. Based on the pretest results, modifications and corrections, like wording, logical sequence, and skip patterns, were made immediately. The questionnaire was translated into Afan Oromo (the local language) for easy understanding and then back-translated into English to ensure consistency. Data collectors and supervisors received 1 day of training covering the aim of the study, data collection methods, questionnaire content, confidentiality protocols, responder's rights, and informed consent before they start the data collection. The overall activity was supervised by the principal investigator of the study.

### Data processing and analysis

The data was coded and entered into EpiData version 3.1. Then, it was exported to SPSS version 27 and cleaned before analysis. Descriptive statistics like mean, median, frequency, and percentage were computed. Bivariate and multivariable logistic analyses were performed to assess the association between categorical variables and knowledge of OF. Variables with *p* < 0.25 in the bivariate analysis were considered for the multivariable logistic regression analysis. The fitness of the logistic regression model was evaluated using the Hosmer–Lemeshow goodness-of-fit test. Multi-collinearity among the independent variables was assessed using multiple linear regression, and the variance inflation factor (VIF) for all variables was found to be less than 10. Finally, the screened variables were fitted to the multivariable logistic regression model using a backward stepwise method to reduce the effects of cofounders and identify the independent effect of each variable on the outcome. Odds ratios with 95% confidence levels and *p*-values < 0.05 were computed to determine the level of significance. The results of the study were presented in narrative form, tables, and figures.

### Ethical consideration

Ethical approval was obtained from the Institute of Research Ethical Review Committee, Hamlin Fistula Ethiopia, with reference number HFE-IRERC 016-2024. A permission letter was obtained from the Ejere Woreda Health Administrative Office. Then, letters of cooperation were also issued to each kebele administration through the Ejere Woreda Health Office. Informed verbal consent was obtained from all study participants. In addition, an affirmation that they were free to withdraw consent and discontinue participation without any form of prejudice was made. Confidentiality of information and privacy of participants were assured.

## Results

### Socio-demographic characteristics

A total of 770 reproductive-age women participated in the study, yielding a response rate of 98.2%. The mean age of participants was 31.35 years (SD = 7.95). The majority, 717 women (93.1%), were married, and 50% identified as Orthodox Christians. The Oromo ethnicity was the most represented, comprising 693 90%) participants.

Regarding education, only 165 women (21.4%) had attended college or higher, while nearly two-thirds, 493 (64%), lived in rural areas. In terms of media exposure, more than half (423 or 54.9%) had access to a television or radio at home, whereas over three-quarters (559, or 77.8%) reported not using the internet (Table 1).

**Table 1:** Socio-demographic characteristics of women of reproductive age in Ejere Woreda, West Shewa Zone, Oromia, Ethiopia, 2024 (*n* = 770).

Variables	Categories	Frequency	Percentage
Age of the participants	15–19	70	9.1
	20–25	235	30.5
	26–30	32	4.2
	≥31	433	56.2
Marital status	Married	717	93.1
	Unmarried	53	6.9
Age at marriage	<18	74	9.6
	≥18	696	90.4
Religion	Orthodox	385	50
	Protestant	310	40.3
	Wakefata	43	5.6
	Muslim	32	4.2
Ethnicity	Oromo	687	89.2
	Amhara	61	7.9
	Others^a^	22	2.9
Women's educational status	Cannot read and write	109	14.2
	Can read and write	218	28.3
	Primary (1–8)	153	19.9
	Secondary (9–12)	125	16.2
	College and above	165	21.4
Women's occupation	Housewife	399	51.8
	Government employee	158	20.5
	Private employee	136	17.7
	Student	77	10
Husband's educational status	Cannot read and write	168	21.8
	Can read and write	78	10.1
	Primary (1–8)	141	18.3
	Secondary (9–12)	153	19.9
	College and above	230	29.9
Husband's occupational status	Government employee	220	28.6
	Private employee	165	21.4
	Daily laborer	58	7.5
	Farmer	327	42.5
Had access to a TV/radio	No	347	45.1
	Yes	423	54.9
Using the Internet (social media)	No	599	77.8
	Yes	171	22.2
Distance from the health facility	<30 min	250	32.5
	≥30 min	520	67.5

^a^ Gurage, Wolayita, or Silte.

### Obstetric characteristics of the participants

A study on pregnancy characteristics revealed that the average age at a woman's first pregnancy was 24.55 years, with a standard deviation of 3.37 years. The majority of participants, 696 women (90.4%), were married by age 18. Notably, two-thirds of the participants, totaling 509 (66.1%) women, received antenatal care (ANC) during their pregnancies. The rates of abortion and stillbirth were relatively low, impacting only 15.8% and 10.1% of the women, respectively.

Regarding delivery locations, most births, 503 (72.5%), occurred in healthcare facilities. However, less than half of the women, specifically 308 (44.4%), received postnatal care (PNC). Family planning practices showed promise, with nearly three-quarters of the women, 567 (73.6%), using modern contraceptives, primarily injectable methods, which accounted for 86.9% of their choices.

While about half of the participants, 395 (51.3%) women, received counseling on obstetric fistula, either during ANC, childbirth, or the postpartum period, a significant proportion, 539 (70%) women, had not attended conferences aimed at pregnant women, which could be considered an obstetric factor related to the occurrence of obstetric fistula (Table 2).

**Table 2:** Obstetrics characteristics of women of reproductive age in Ejere Woreda, West Shewa Zone, Oromia, Ethiopia, 2024 (*n* = 770).

Variables	Categories	Frequency	Percentage
Gravidity	Nulligravida	76	9.9
	Primigravida	78	10.1
	Multigravida	616	80.0
ANC contact for the recent pregnancy	No	261	33.9
	Yes	509	66.1
Number of ANC contacts (*n* = 539)	<5	231	42.9
	≥6	308	57.1
Place of delivery (*n* = 694)	Health facility	503	72.5
	Home	191	27.5
Reason(s) for home delivery (*n* = 191)	Lack of transport	39	25.0
	Lack of women's decision-making power	21	13.5
	Poor road condition	10	6.4
	No facility nearby	37	23.7
	No privacy at health facilities	41	21.5
	Others^a^	8	4.2
Mode of delivery (*n* = 694)	Spontaneous vaginal delivery	457	65.9
	Operative vaginal delivery	111	16.0
	Cesarean delivery	126	18.2
Who conducted delivery (*n* = 694)	TBA	156	22.5
	Healthcare provider	538	77.5
Counsel about OF	Yes	395	51.3
	No	305	48.7
PNC for the last birth (*n* = 694)	No	386	55.6
	Yes	308	44.4
Family planning use	No	203	26.4
	Yes	567	73.6
Types of contraceptives used (*n* = 567)	Pills	68	8.8
	Implants	493	64.0
	Injectables	197	25.6
	IUCDs	53	6.9
	Others^b^	29	5

TBA, traditional birth attendant; IUCD, intrauterine contraceptive device.

^a^ Sudden onset of labor, culture, or influence of family members.

^b^ Condom or post-pill.

### Knowledge of women about obstetric fistula

**Figure 2:**
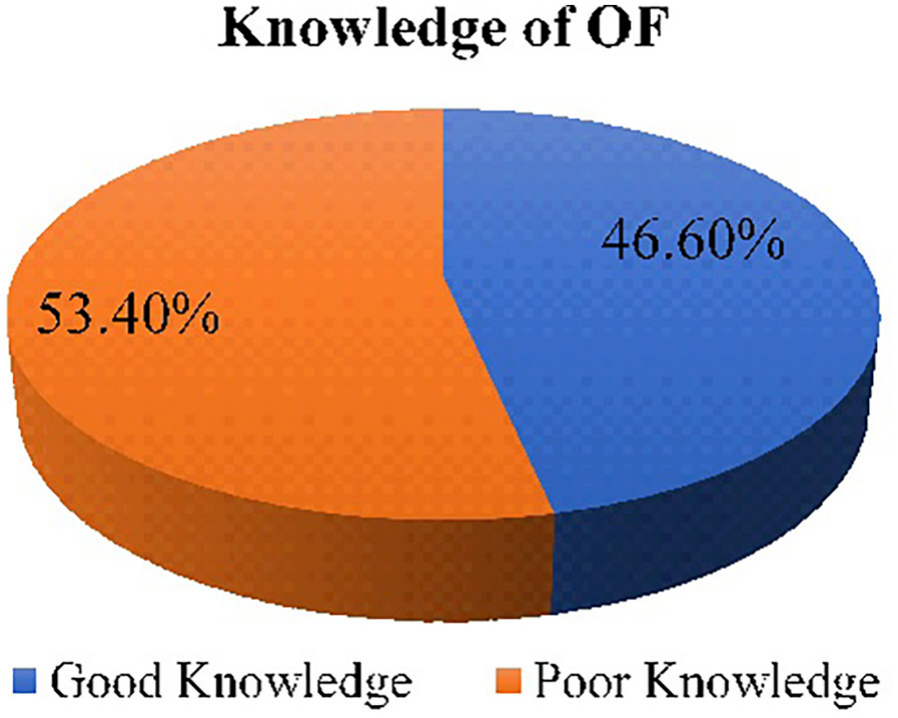
Overall knowledge of OF among reproductive-age women in Ejere Woreda, West Shewa Zone, Oromia Region, Ethiopia, 2024 (*n* = 770).

In this study, 46.6% of reproductive-age women demonstrated knowledge about obstetric fistula [95% confidence interval (CI): 43.1–50.3] (Figure 2).

**Figure 3:**
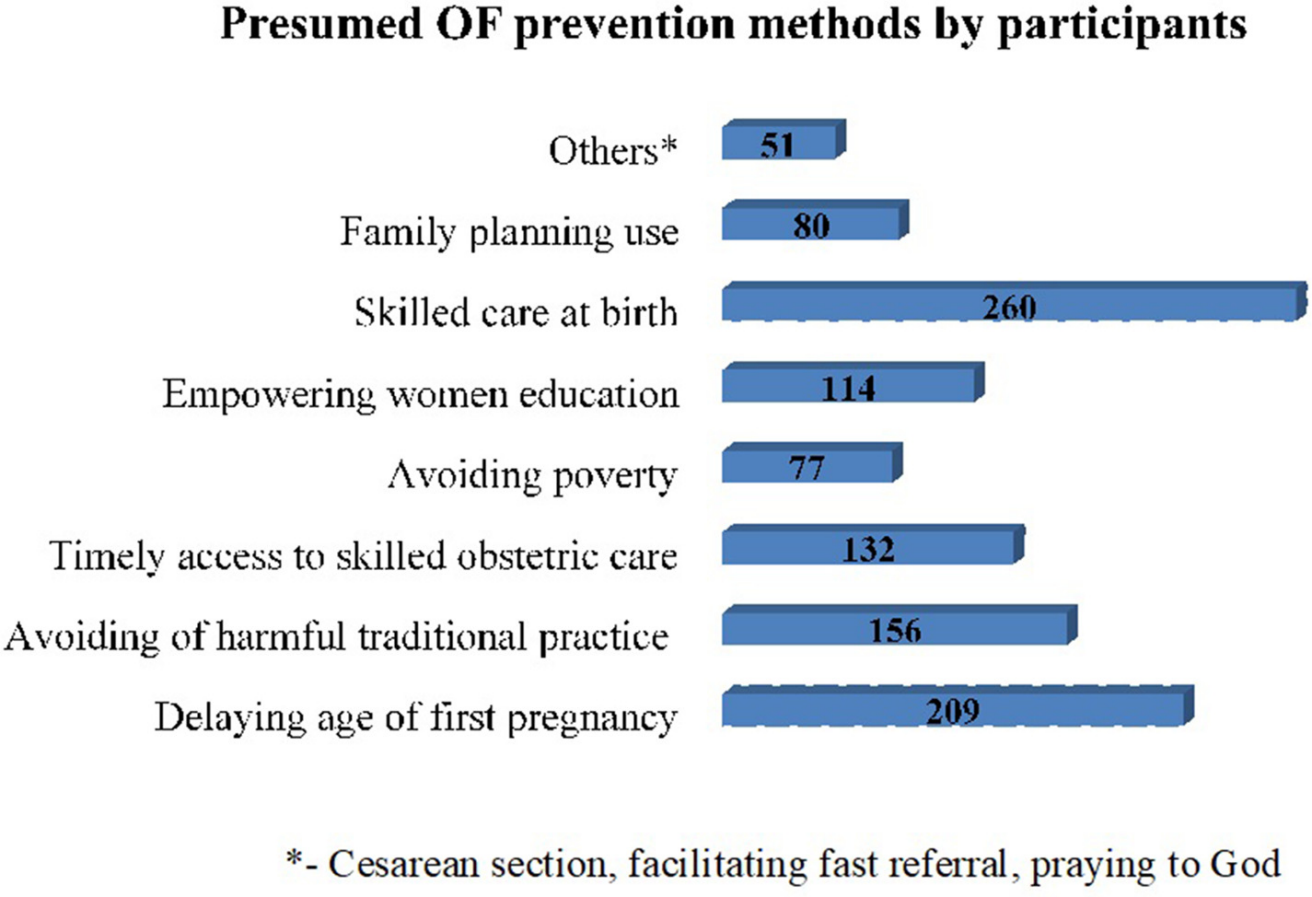
Mentioned prevention methods of obstetric fistula by women of reproductive age in Ejere Woreda, central Ethiopia, 2024.

The majority of the participants, 616 (80%), were aware of OF; among them, 539 (87.5%) received information from healthcare providers, and 498 (80.8%) from TV/radio. Participants demonstrated some understanding of symptoms, commonly citing foul-smelling vaginal discharge (388, 87.8%) and urinary incontinence (337, 76.2%). Regarding risk factors, obstructed labor (263, 61.4%) and prolonged labor (228, 53.3%) were frequently mentioned (Table 3). Encouragingly, a significant portion of women identified skilled birth attendance (260, 74.3%) and delaying first pregnancy (209, 59.7%) as preventive measures (Figure 3).

**Table 3:** Knowledge about obstetric fistula among women in Ejere Woreda, West Shewa Zone, Oromia, Ethiopia, 2024 (*n* = 770).

Variables	Categories	Frequency	Percentage
Ever heard of OF?	No	154	20.0
	Yes	616	80.0
Source of information (*n* = 616)	Healthcare providers	539	87.5
	School	230	37.3
	TV/radio	498	80.8
	Family	77	12.5
	Friends	348	56.5
	Victim of OF	118	19.2
Know the type of OF?	No	462	60.0
	Yes	308	40.0
Types of OF you know (*n* = 308)	Recto-vaginal fistula	92	29.9
	Vesico-vaginal fistula	126	40.9
	Both	90	29.2
Know the signs/symptoms of OF?	No	328	42.6
	Yes	442	57.4
Signs/symptoms of OF you know (*n* = 442)	Urinary incontinency	337	76.2
	Fecal incontinency	216	48.9
	Vulvar irritation	105	23.8
	Foul-smelling vaginal discharge	388	87.8
	Leakage of gas or flatus	125	28.3
	Dyspareunia	166	37.6
	Others^a^	154	34.8
Know the causes/risk factors of OF?	No	342	44.4
	Yes	428	55.6
Causes/risk factors of OF you know (428)	Prolonged labor	228	53.3
	Obstructed labor	263	61.4
	Childhood malnutrition	63	14.7
	Operative vaginal delivery	56	13.1
	Early marriage	161	37.6
	Younger age at first delivery	131	30.6
	Home delivery	205	47.9
	Unspaced birth	170	39.7
	Lack of obstetric care	201	46.9
	Big baby	28	6.5
	Others^b^	21	4.9
Is OF preventable?	No	420	54.5
	Yes	350	45.5
Is OF a treatable problem?	No	231	30.0
	Yes	539	70.0
Type of treatment (*n* = 539)	Medical	232	43.2
	Surgical	117	21.7
	Traditional medicines	47	0.09

^a^ Social isolation, depression, or embarrassment.

^b^ Illiteracy, delayed arrival at a health facility for delivery, or delayed referral system.

### Factors associated with knowledge about obstetric fistula

After controlling for potential confounders in a multivariable logistic regression, several factors were significantly associated with women's knowledge of OF at a *p*-value of <0.005.

Women living in urban areas were 4.12 times more likely to understand OF compared to those in rural areas [adjusted odds ratio (AOR) = 4.12, 95% CI: 2.36–7.19]. Access to a TV or radio at home increased the likelihood of good knowledge about OF by 2.51 times (AOR = 2.51, 95% CI: 1.19–5.25). In addition, women whose homes were within a 30-min travel distance from health facilities were 4.88 times more likely to have a solid understanding of OF (AOR = 4.88, 95% CI: 2.37–10.04).

Women who delivered at healthcare facilities had an increased likelihood of good OF knowledge, being 4.62 times more informed than those who did not (AOR = 4.62, 95% CI: 2.56–8.33). Participation in pregnant women's conferences also played a significant role; those who attended were 3.42 times more likely to have good knowledge of OF compared to those who did not (AOR = 3.42, 95% CI: 1.88–6.22). Finally, women with a history of modern contraceptive use were nearly five times more likely to understand OF than those who had never used them (AOR = 4.82, 95% CI: 2.77–8.37) (Table 4).

**Table 4:** Factors associated with knowledge of obstetric fistula among women in Ejere Woreda, West Shewa Zone, Oromia Region, Ethiopia, 2024(*n* = 770).

Variables	Categories	Knowledge of OF		COR (95% CI)	AOR (95%, CI)	*p*-value
		Good (%)	Poor (%)			
Women's occupation	Housewife	123 (30.8)	276 (69.2)	1	1	1
	Government employee	112 (70.9)	46 (29.1)	5.46 (3.65–8.18)	1.07 (0.52–2.19)	0.859
	Private business	70 (51.5)	66 (48.5)	2.38 (1.59–3.54)	0.19 (0.09–0.45)	0.100
	Student	23 (29.9)	54 (70.1)	5.27 (3.09–8.97)	3.59 (0.01–1.0)	0.610
Place of residency	Urban	220 (69.8)	95 (30.2)	5.27 (3.85–7.19)	**4.12 (2.36–7.19)**	**0**.**001**
	Rural	139 (30.5)	316 (69.5)	1	1	1
Have access to a TV/radio	Yes	264 (62.4)	159 (37.6)	4.40 (3.24–5.99)	**2.51 (1.19–5.25)**	**0**.**015**
	No	95 (27.4)	252 (72.6)	1	1	1
Using the internet	Yes	133 (77.8)	38 (22.2)	5.78 (3.89–8.59)	1.84 (0.87–3.88)	0.109
	No	226 (37.7)	373 (62.3)	1	1	1
Distance from health facilities	<30 min	186 (74.4)	64 (25.6)	5.83 (4.16–8.17)	**4.88 (2.37–10.0)**	**0**.**001**
	≥30 min	173 (33.3)	347 (66.7)	1	1	1
ANC contacts	Yes	273 (53.6)	236 (46.4)	2.35 (1.72–3.21)	1.69 (0.96–2.97)	0.068
	No	86 (33)	175 (67)	1	1	1
Place of delivery	Health facility	256 (50.9)	247 (49.1)	2.92 (2.03–4.22)	**4.62 (2.56–8.33)**	**0**.**001**
	Home	50 (26.2)	141 (73.8)	1	1	1
Family planning use	Yes	307 (54.1)	260 (45.9)	3.43 (2.40–4.89)	**4.82 (2.77–8.37)**	**0**.**001**
	No	52 (25.6)	151 (74.4)	1	1	1
Number of ANC contacts	<5	142 (30.7)	320 (69.3)	5.37 (3.92–7.36)	1.31 (0.67–2.59)	0.431
	≥6	217 (70.5)	91 (29.5)	1	1	1
Attended pregnant women's conference	Yes	154 (66.7)	77 (33.3)	3.26 (2.36–4.51)	**3.42 (1.88–6.22)**	**0**.**01**
	No	205 (38.0)	334 (62.0)	1	1	1

COR, crude odds ratio.

Bold indicates significant variables.

## Discussion

In this study, an attempt has been made to assess the level of knowledge of OF and its associated factors among reproductive-age women in Ejere Woreda, Oromia Region, central Ethiopia, where the result indicated that 46.6% (95% CI: 43.1–50.3) of participants had good knowledge of OF. Socio-demographic factors like place of residence, access to TV/radio, and proximity to healthcare facilities significantly influenced OF knowledge. In addition, place of delivery during the last pregnancy, history of modern contraceptive use, and attending pregnancy conferences were also significant predictors of OF knowledge.

The current study found that the overall prevalence of OF knowledge was 46.6% (95% CI: 43.1–50.3). The finding is in line with studies conducted in northern Ghana (24) and southwest Ethiopia (20), which reported knowledge levels of 45.8% and 50%, respectively. However, it is lower than findings from two studies in Nigeria, where 57.8% (25) and 52% (26) of respondents demonstrated a good level of OF knowledge. It is also lower than the result from a study in Tanzania, which reported a knowledge level of 60% (27). The variation may stem from differences in healthcare infrastructure, educational campaigns, and community outreach. In addition, cultural attitudes toward women's health, educational levels, and the focus of local health policies could contribute to the disparities in knowledge levels observed. Conversely, this finding is higher than that reported in most of the studies conducted in Ethiopia (18, 19, 28, 29). The variation in knowledge levels could be due to the progress in public health education, the expansion of awareness campaigns, and enhanced healthcare outreach programs. Renovation of local or national health policies, better access to maternal health services, and increased community engagement in health education could have contributed to this improvement.

Women living in urban areas were found to be 4.12 times more likely to be knowledgeable about obstetric fistula than those in rural areas. This finding aligns with earlier research from Ethiopia (18, 28) and Burkina Faso (30). The higher awareness in urban areas is likely due to greater access to information and education through healthcare facilities, media, and community networks, which increase exposure to key health messages about OF.

Access to television or radio also plays a significant role in raising awareness. Women who had access to TV or radio at home were 2.51 times more likely to have good knowledge of obstetric fistula than those who did not. This finding is supported by previous studies from Ethiopia (16, 18) and Gambia (31). The likely reason is that these media platforms provide women with vital information on OF, including its causes, symptoms, and available treatment options.

Another strong predictor of knowledge was proximity to healthcare facilities. Women living within a 30-min travel distance to a health center were 4.88 times more likely to be well-informed about OF compared to those residing farther away. This observation is consistent with prior studies conducted in Ethiopia (16, 32). A possible justification could be that women living closer to health facilities are more likely to utilize maternal healthcare services, such as ANC, delivery, and PNC (33–35). This increased interaction with the healthcare system provides women with opportunities to receive education and counseling about OF. However, challenges such as travel costs, time constraints, and reduced access to health education, which are commonly experienced in remote areas, could reduce awareness and understanding of OF.

In addition, the place of childbirth was found to influence knowledge levels. Women who delivered in health facilities were 4.62 times more likely to have good knowledge about obstetric fistula than those who gave birth at home. Similar findings have been reported in studies from Afghanistan (36) and Ethiopia (28–29, 37). Institutional deliveries often provide greater opportunities for women to receive health education, including information about OF, through interactions with healthcare professionals and exposure to educational materials such as posters and visual aids.

To reduce the urban–rural disparity in OF knowledge and improve access to health information in underserved areas, targeted interventions are essential. These should include culturally appropriate community education, enhanced maternal health services, and customized communication strategies. Integrating OF awareness into reproductive health programs, strengthening the training of health extension workers with a focus on fistula, and utilizing locally accessible media, especially TV and radio, can help disseminate crucial messages and improve prevention and early detection efforts in rural areas.

Our study identified participating in pregnant women's conferences during pregnancy as a predictor of OF knowledge. Accordingly, women who attended these conferences were 3.42 times more likely to have good knowledge compared to those who did not. Previous studies conducted in Ethiopia also reported similar findings (28, 32, 37). This could be attributed to the fact that conferences often include sessions and presentations aimed at raising awareness about OF and other maternal health issues. The interactive nature of these events allows women to engage directly with healthcare professionals and ask questions, further enhancing their understanding of the condition. Furthermore, conferences serve as a platform where peer-to-peer learning modalities are carried out, enabling the dissemination of health information and the sharing of experiences related to OF.

Finally, the study found that women with a history of modern contraceptive use were 4.82 times more likely to have good knowledge of OF compared to those had not used contraceptives. The same result was reported in a study conducted in the southeastern Tigray Region of Ethiopia (29). This might be because women who use modern contraceptives are more likely to have interactions with healthcare providers, which provide them with opportunities to receive health education, including information about maternal health complications like obstetric fistula. A woman attending a family planning service may receive counseling on pregnancy spacing, danger signs during pregnancy, and complications like fistula. This increases her knowledge level of OF compared to someone without contact with healthcare providers.

### Strengths and limitations of the study

The use of a community-based study design, which included a reasonably representative sample size and a comprehensive assessment of various factors associated with women's knowledge of obstetric fistula, truly estimated the knowledge level of obstetric fistula within the population and also offered valuable insights to inform targeted interventions aimed at improving awareness. Nevertheless, the cross-sectional study design restricts the ability to establish causal relationships, and dependence on self-reported data may introduce recall bias.

## Conclusion and recommendations

The study identified urban residence, access to media (TV/radio), proximity to healthcare facilities, history of institutional delivery, modern contraceptive use, and participation in pregnancy conferences as significant predictors of knowledge about OF. The findings of this study underscore the critical need to bridge the urban–rural disparity in healthcare access and information dissemination to improve knowledge of obstetric fistula and preventing its occurrence. A multifaceted approach that combines infrastructural improvements, media engagement, community education, and the integration of OF awareness into existing maternal health services is essential. By implementing these targeted strategies, policymakers and health practitioners can significantly reduce the burden of obstetric fistula and improve maternal health outcomes across diverse communities.

## Data Availability

The raw data supporting the conclusions of this article will be made available by the authors, without undue reservation.
